# Leaflet-specific effects of charged lipids on a voltage-gated potassium channel

**DOI:** 10.1016/j.jlr.2025.100934

**Published:** 2025-11-03

**Authors:** Takahisa Maki, Masako Takashima, Masayuki Iwamoto, Shigetoshi Oiki

**Affiliations:** 1 Faculty of Medical Sciences, Deptartment of Molecular Neuroscience, University of Fukui, Fukui, Japan; 2 Biomedical Imaging Research Center, University of Fukui, Fukui, Japan

**Keywords:** membrane leaflet, voltage-dependent gating, potassium channel, contact bubble bilayer, asymmetric membrane, surface potential, intramembrane potential, functional domain

## Abstract

Voltage-gated potassium (Kv) channels possess distinct voltage-sensor (VSD) and pore (PD) domains, making it challenging to study domain-specific lipid effects. Here, we examined the functional modification of a prototypical Kv channel, KvAP, by phosphatidylglycerol (POPG) and phosphatidylserine (POPS) in mono-component asymmetric bilayers using the contact bubble bilayer (CBB) method. In these membranes, specific chemical modifications were distinguished from non-specific electrostatic (surface potential) effects by using the channel's gating as an intrinsic probe. No specific effects were observed when charged lipids were in the outer leaflet. When present in the inner leaflet, POPS exerted only a single specific effect: the acceleration of activation kinetics. In contrast, inner-leaflet POPG induced multiple, profound modifications: it also accelerated activation kinetics, but additionally shifted the conductance-voltage (*G*-*V*) curve hyperpolarized, attenuated the G-V slope, and accelerated inactivation kinetics. This clear contrast allows a domain-specific interpretation: the shared acceleration of activation is attributed to a general kinetic modulation of the VSD, while POPG’s unique effects—impaired electromechanical coupling (attenuated slope) and accelerated inactivation—are attributed to specific chemical interactions with the VSD-PD linker and the PD, respectively. These results reveal a multi-site mechanism of lipid modulation dictated by leaflet asymmetry and headgroup chemistry.

## Graphical abstract

**Figure:**
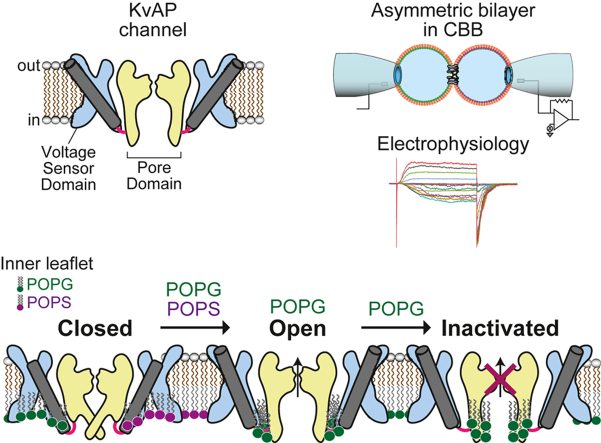

Cell membranes comprise a variety of lipids with spatially inhomogeneous distributions (1, 2), and membrane proteins are surrounded by continuously changing lipid species, which modify their function. Some lipids occupy specific sites on the membrane protein surface, whereas others transiently hit proteins (3). A broad spectrum of dynamic lipid–protein interactions encompasses non-annular to annular lipids, including those in rafts (4, 5, 6). Moreover, cell membranes are asymmetric in their constituents, and different chemical species of membrane lipids render distinct physical features to the leaflets (1, 7). Consequently, studying the dynamic and functional interplay between specific lipid species and membrane proteins is challenging because of the spatial and temporal inhomogeneity of the cell membrane (8, 9, 10, 11).

Voltage-gated potassium (Kv) channels play a central role in action potential propagation in neuronal cell membranes by modifying the repolarization process (12). Without Kv channels, the repetitive and frequent generation of action potentials is not feasible (13). Various Kv channels exhibit altered gating activity by membrane lipids (14, 15, 16, 17, 18, 19). Accordingly, the lipid composition around channel proteins in neuronal membranes should control action potential propagation, which is, however, elusive for in situ experiments. This situation is more complicated for Kv channels. The structure of Kv channels has a distinct domain for gated ion permeation (pore domain [PD]) surrounded by four voltage-sensing domains (voltage-sensor domain [VSD]) (Fig. 1A). Each domain has a compact structure (20, 21, 22) and is surrounded by different types of lipids in the cell membranes, determined by specific lipid–domain interactions (22, 23). Thus, functional changes in each domain in various lipid environments modify the frequency of action potential repetitions, where the lipid environment for each domain in the cell membrane has not been experimentally controlled.

Here, we studied the effects of membrane anionic lipids on a prototypical Kv channel, KvAP, from archaebacteria, which exhibits similar functional features in voltage-dependent gating with a homologous transmembrane structure with a short amino acid length (approximately 300) relative to its eukaryotic counterparts (24). Anionic lipids, phosphatidylglycerol (POPG) and phosphatidylserine (POPS), have various effects on channel activity (14, 25, 26, 27, 28). We examined these lipids in synthetic lipid bilayers with defined lipid compositions to determine lipid domain interactions (29, 30). Unlike biological processes generating membrane asymmetry through active transport by flippase and floppase (31), the contact bubble bilayer (CBB) method generates de novo synthesis of asymmetric membranes by mechanically docking two separately prepared leaflets (32, 33, 34), with each leaflet containing single lipid constituents (mono-component leaflets) (7, 29) (Fig. 1B). Accordingly, both the VSD and the PD domains of the Kv channels are surrounded exclusively by a single lipid species in the outer and inner halves of the membrane-embedded surfaces. Electrophysiological characterization can address the functionally modified features of voltage-dependent gating of the KvAP channel, whether they are specific to PD or VSD. A key challenge when studying charged lipids is to discriminate their specific chemical interactions from their non-specific physical effects. The presence of charged lipids in an asymmetric membrane generates a surface potential, which in turn alters the intramembrane potential experienced by the channel's voltage sensor. This electrostatic influence can shift the apparent voltage dependence of gating. Our approach allows for the precise quantification of this channel-sensed potential shift, enabling its rigorous separation from true chemical modifications.

Systematic experiments with varied asymmetric membranes revealed a specific effect of POPG on the KvAP channel. We focused on the effects of lipid species on a channel protein in two respects: (i) the sidedness of either outer or inner annular lipids and (ii) two distinct functional domains of either VSD or PD, which were studied in simple mono-component leaflets. This two-pivot approach is generally valid for studying multidomain membrane proteins with varied membrane lipid compositions.

## Materials and methods

### Reagents

The phospholipids used in the KvAP study were 1-palmitoyl-2-oleoyl-sn-glycero-3-phospho-(10-rac-glycerol) (sodium salt) (16:0–18:1 PG, POPG), 1-palmitoyl-2-oleoyl-sn-glycero-3-phospho-L-serine (sodium salt) (16:0–18:1 PS, POPS) and 1,2-diphytanoyl-*sn*-glycero-3-phosphocholine (4ME 16:0 PC, DPhPC). These lipids were purchased from Avanti Polar Lipids. All other chemicals were purchased from Nacalai Tesque.

### KvAP cloning, expression, and purification

DNA encoding residues 14–295 of KvAP (uniport ID: Q9YDF8) fused with the thrombin recognition site and a 6×His tag was codon-optimized for *Escherichia coli* and synthesized by GenScript. The optimized KvAP DNA was cloned into the pET29(a) plasmid using an In-Fusion cloning Kit (639648, Takara Bio). The DNA fragments for cloning were amplified using PrimeStar Max DNA polymerase (R045A, Takara Bio) and the primer sets for the KvAP fragment (5′-TTTGTTTAACTTTAAGAAGGAGATTAACCATGGCCCGCTTTCG-3′ and 5′-TCCTTTCGGGCTTTGTTAGTGATGGTGATGGTGATGAGATC-3′) or the plasmid (5′-CAAAGCCCGAAAGGAAGCTG-3′ and 5′-TCCTTCTTAAAGTTAAACAAAATTATTCTAGAGGGGAATTGTTA-3′).

KvAP-His protein was expressed in *E. coli* BL21 (DE3) as follows: Cells harboring the KvAP expression plasmid were cultured at 37°C until the optical density at 600 nm (OD_600_) reached 0.6 in Luria broth (LB) medium containing kanamycin. IPTG was added to a final concentration of 0.2 mM, and the culture was further incubated for 3 h at 37°C. The cells were collected by centrifugation, and the pellet was suspended in S buffer (20 mM HEPES-KOH [pH 7.4], 200 mM KCl). The purification method was as per Lee *et al.* (35) with the following modifications: The cell pellet expressing KvAP was resuspended in S buffer (5 ml per 1 g of cells) containing PMSF (0.5 mM) and disrupted by sonication. After adding dry powder of n-Decyl-β-D-maltoside (DM; D382, Dojindo) to a final concentration of 2% (w/v), the cell lysate was rotated for 3 h at room temperature. Insoluble materials were removed by ultracentrifugation at 200,000 × g for 30 min at 4°C. The supernatant was mixed with Talon-resin (635652, Takara Bio) (1 ml resin slurry per 20 ml of lysate) at 4°C for 1 h with agitation. The resin was transferred to a pre-washed Bio-spin column (7321010, Bio-Rad) and washed with 10 bed volumes of wash buffer (10 mM HEPES-KOH [pH 7.4], 200 mM KCl, 0.25% (w/v) DM, 25 mM imidazole). The KvAP protein was then eluted in five bed volumes of elution buffer (10 mM HEPES-KOH [pH 7.4], 200 mM KCl, 0.25% (w/v) DM, and 500 mM imidazole). The pooled fraction was concentrated using an Amicon Ultra-4 (50 k) centrifugal filter (UFC805008, Merck Millipore). The concentrated solution was then developed in a Superdex 200 Increase 10/300 Gl gel filtration column (28990944, Cytiva) in a gel filtration buffer (10 mM HEPES-KOH [pH 7.4], 200 mM KCl, and 0.25% (w/v) DM).

### Reconstitution of KvAP into liposomes

KvAP-reconstituted liposomes were prepared by dialysis. The mixture solution containing 0.1 mg/ml KvAP, 5 mg/ml DPhPC (850356C, Avanti), POPS (840034C, Avanti) or POPG (840457C, Avanti), 10 mM HEPES-KOH [pH 7.4], 200 mM KCl, 0.1% (w/v) DM, and 1% (w/v) n-Octyl-β-D-glucoside (25543-14, Nacalai Tesque) was loaded into a dialysis tube (132650, REPLIGEN) and placed into the dialysis buffer (10 mM HEPES-KOH [pH 7.4], 200 mM KCl). The dialysis buffer was changed five times every 12 h. The final liposome suspension contained 10 mM HEPES (pH 7.4) and 200 mM KCl, with lipid concentrations of 5 mg/ml (DPhPC, POPS or POPG) and 0.1 mg/ml KvAP.

### Contact bubble bilayer (CBB) experiments

The CBB method used in this study has been previously described (32, 33, 36). Glass pipettes for the CBB experiments are borosilicate glass capillary (OD/ID; 1.50/1.05 mm). The tips of the pipettes were made using a micropipette puller (P-87, Sutter Instrument, Novato, CA, USA) and broken with a micro-forge (MF-830, Narishige, Tokyo, Japan) to achieve a diameter of 30 μm. The liposome or proteoliposome with desired lipid composition was loaded into the pipette, containing 200 mM KCl electrolyte, and the pH was 7.4, buffered by HEPES. The pipettes were manipulated using a motorized micromanipulator (EMM-3NV, Narishige) under an inverted microscope (IX73, Olympus, Tokyo, Japan). The pipette pressure was controlled using a pneumatic microinjector (IM-11-2, Narishige).

A glass slide with a concave depression was placed on a microscope stage, and a small amount of hexadecane (200 μl) was filled in the concave (hexadecane bath). The tips of the two pipettes were immersed in a hexadecane bath. A water-in-oil bubble was inflated into the bath from the pipette tip by applying pressure to the pipette solution. A bubble with a radius of 30 μm was maintained using a pressure controller (36), and a phospholipid monolayer was spontaneously formed at the oil-water interface. Two bubbles are docked by micromanipulators, forming a contact bubble bilayer (CBB).

**Fig. 1:**
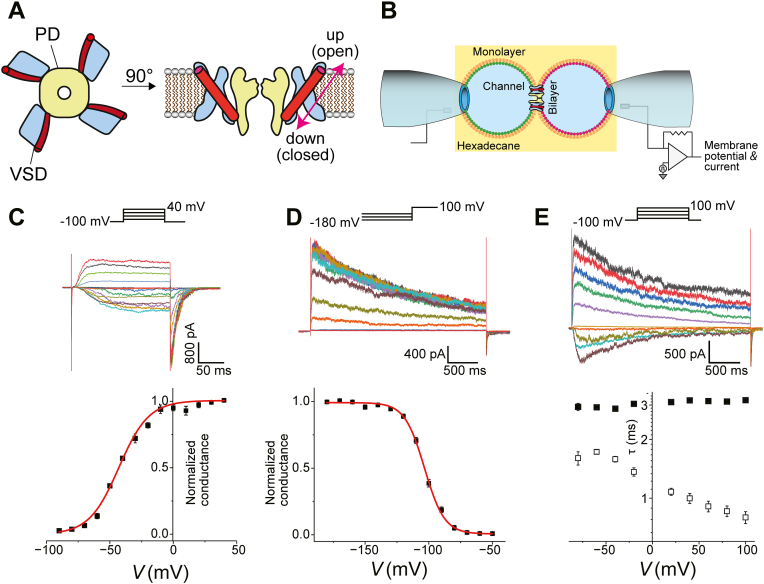
Voltage-gated activities of the KvAP channel in symmetric DPhPC membrane. A: Schematic representation of the KvAP channel. Top (left) and side (right) views of the model. The PD (yellow) is surrounded by four VSDs (cyan), each with a positively charged S4 helix (red). When S4 in all four VSDs is in the up configuration, the gate in the PD opens. B: Experimental configuration of the CBB. A monolayer on the bubble surface was formed from liposomes suspended in an aqueous bubble solution. Each bubble lined with a distinct monolayer was docked to form a bilayer. In the case of asymmetric membrane formation, the left and right bubbles contained different lipid species as liposomes, such as POPG in the left bubble and DPhPC in the right bubble. KvAP channels are added as KvAP-reconstituted liposomes in the right bubble, which are spontaneously incorporated into to a bilayer. For electrophysiological studies, the membrane potential was defined as the right side relative to the left side as the ground. C: Voltage-dependent activation of the KvAP channel. Upper: Current traces for short (200 ms) depolarizing pulses (upper inset) exhibiting voltage-dependent activations after delays (activation currents) and rapid deactivation upon repolarization (tail currents). The membrane potential was depolarized (−90 to +40 mV) from a holding potential of −100 mV and repolarized to −100 mV. Lower: The conductance-voltage (*G-V*) curve, fitted with the Boltzmann function (see Methods), having *V*_half_^Act^ of −44.56 ± 1.82 mV and *s*^Act^ of −2.52 ± 0.16/mV (N = 5). D: Steady-state inactivation. Currents were fully inactivated by depolarization pulses of +100 mV for 3s, which was recovered more or less by retention at hyperpolarized potentials (−180 to −50 mV) for 15 s. Current traces shown elicited current by depolarization pulses of +100 mV, which were recovered from the inactivation during long hyperpolarized potentials. Lower: The steady-state inactivation curve, which was fitted with the Boltzmann function. The *V*_half_^Inact^ was −102.86 ± 0.69 mV, and *s*^Inact^ was 3.24 ± 0.26/mV (N = 5). E: Voltage-dependent slow inactivation of the KvAP channel upon long (3000 ms) depolarizing pulses. Upper panel: Current traces for long depolarizing pulses. The membrane potential was depolarized (−80 to +100 mV) from the holding potential of −100 mV. The current traces were fitted for the time constants (see Methods). Lower panel: Voltage-dependent time constants for activation (τ^Act^, open symbols) and inactivation (τ^Inact^, closed symbols).

In the asymmetric bilayer formation, the left bubble contained liposomes of POPG or POPS (37), whereas the right bubble contained liposomes of DPhPC) (Fig. 1B). Accordingly, the monolayer formed at the oil-water interface on the left bubble was composed exclusively of POPG or POPS, whereas the monolayer on the right bubble was composed of DPhPC. Upon docking of two bubbles, a purely asymmetric bilayer is formed, whose asymmetry is maintained unless the bubbles are unbroken (7, 32, 33, 34). When the bubbles were broken, the procedure was started from the beginning by filling the pipette solution. KvAP channels were reconstituted to the bilayer by adding proteoliposomes into the right bubble.

### Single- and macroscopic current recordings of the KvAP channel

Single- and macroscopic-channel currents were recorded using an analog amplifier (Axopatch 200B, Molecular Devices, USA). A four-pole Bessel filter with a cutoff frequency (*f*_c_^Bessel^) of 2 kHz (−3 dB) was used, and the sampling frequency was set to 10 kHz. For depolarizing pulses, the membrane potential was set to −200 mV after returning to −100 mV to recover possible inactivation. When the amplitude of the macroscopic currents was small, the current traces were ensembled for macroscopic current characterization.

When massive channels were reconstituted into a CBB, the currents were examined using short depolarization pulses. In one membrane, macroscopic currents were activated exclusively at positive potentials, whereas in the other membrane, macroscopic currents were activated at negative potentials. These results indicate that the channels were reconstituted into a CBB with the same orientation, whereas their orientation was determined by chance for each CBB.

The membrane potential was initially defined as the potential of the right bubble, with the left bubble serving as ground. To standardize the analysis, the polarity of the command voltage was inverted for channels that were activated at negative potentials. The detailed logic for assigning the channel orientation based on this procedure is described in the Results section.

### *G*-*V* curve

The *G–V* curves were evaluated using peak currents at depolarized potentials. The peak current at a voltage (*I*_peak_(*V*)) was corrected for a non-linear single-channel current-voltage relationship (*i*(*V*)), such that *I*_peak_(*V*)/*i*(*V*), which was normalized to the value at +40 mV.

### Steady-state inactivation curve

The steady-state inactivation curve was obtained by the following voltage protocol. The current was first inactivated by applying a 3 s depolarization pulse of +100 mV. Then, the membrane potential was hyperpolarized (typically, −180 to −40 mV) for 15 s, during which the inactivated channels were more or less recovered from inactivation. The degree of recovery was evaluated by applying depolarization steps to +100 mV, where the peak current amplitude was measured. The peak current as a function of the hyperpolarized voltage was plotted (steady-state inactivation curve).

### Boltzmann fit for the *G*-*V* and steady-state inactivation curves

The Boltzmann fit was performed using the following function:

$$P\left(V\right)=\frac{1}{1+\mathrm{Exp}\left[-s\;\left(V-V_{half}\right)\right]}$$

where *V*_half_ is the half-activation or -inactivation voltage, and *s* is the slope factor.

### Hodgkin-Huxley model fitting

The kinetics of the voltage-clamped KvAP channels were described based on the Hodgkin-Huxley model with single exponential inactivation (38).

$$I\left(t\right)=A{\left(1-\mathrm{Exp}\left[-t/\tau_{act}\right]\right)}^{4}\left\{h_{0}-\left(h_{0}-h_{\inf}\right)\left(1-\mathrm{Exp}\left[-t/\tau_{inact}\right]\right)\right\}$$

where A is the amplitude, τ_act_ and τ_inact_ are the activation and inactivation time constants, respectively, and *h*_0_ and *h*_inf_ are the inactivation *h* values at the start and infinity, respectively.

Current traces for long depolarization pulses were fitted with the equation.

### Quantification of the channel-sensed potential shift

Charged lipids generate a surface potential at the membrane interface, which alters the electric field within the membrane (12). Consequently, the intra-membrane potential (*V*_intramemb_) experienced by the channel's voltage sensor is offset from the applied transmembrane potential (*V*_m_) by a potential shift, Δ*V*. As voltage-gated channels respond exclusively to *V*_intramemb_, a purely physical, electrostatic effect must manifest as a simple parallel shift of the voltage-dependent curves, without altering the intrinsic voltage sensitivity (the slope factor, *s*) (39).

This principle allows the channel's own gating to serve as a direct probe for quantifying the potential shift, Δ*V*, that it senses. We determined the optimal value of Δ*V* by finding the single Δ*V* value that best reconciled the voltage-dependent curves from the asymmetric membranes with the control (symmetric DPhPC) curves, based on the assumption that a purely electrostatic effect would induce shifts of equal magnitude and opposite sign (+Δ*V* for '-out' and -Δ*V* for '-in').

For the asymmetric POPS membranes, which exhibited no specific effects on gating thermodynamics (i.e., no change in slope factors), we performed a global fitting procedure. The *G*-*V* and steady-state inactivation curves from both the PS-out and PS-in conditions were simultaneously fitted to the respective control curves, with Δ*V* as the single free parameter. This robust procedure yielded the empirically determined potential shift for POPS (Δ*V*^PS^).

For the POPG membranes, this approach could not be applied directly because the PG-in condition showed a significant change in the *G*-*V* slope, violating the parallel-shift assumption. Therefore, we estimated Δ*V*^PG^ using only the data from the PG-out condition, which did exhibit a simple parallel shift consistent with a purely electrostatic effect.

This empirically determined Δ*V* was then used to calculate the effective intra-membrane potential (*V*_intramemb_ = *V*_m_ ± Δ*V*) for all conditions, allowing for the isolation and analysis of specific chemical interactions.

### Statistical evaluation

Voltage-dependent parameters, such as the *G–V*, steady-state inactivation, and τ-*V* curves, were quantitatively examined. The errors in the statistical data are the standard error of the mean (SEM), and the number of data points is shown as N. P values were calculated using Tukey’s honest significant difference (HSD) test after an initial analysis of variance (ANOVA). Significant differences are indicated by ∗ when *P* < 0.01. All numerical, fitting, and statistical calculations were performed using Mathematica.

## Results

### Voltage-dependent gating of the KvAP channel in symmetric membrane

We first examined the gating behavior of the KvAP channel (Fig. 1A) in a symmetric membrane formed with diphytanoylphosphatidilcholine (DPhPC) in the CBB system as a control experiment (Fig. 1B) (14, 25). KvAP channels from proteoliposomes were spontaneously reconstituted into the CBB membrane (see Methods). Voltage-gated activation of macroscopic currents was examined using a series of short depolarization pulses of 200 ms from a holding potential (*V*_hold_) of −100 mV (Fig. 1C, upper). Capacitance transients to voltage steps are instantaneous owing to the small membrane area compared to that of conventional planar lipid bilayers (14). The KvAP channel activated after a delay with a sigmoidal time course, where the channel remained active during 200 ms depolarization pulses over the entire voltage range of −90 to +40 mV. The conductance–voltage (*G–V*) curve is shown in Figure 1C, lower, which was fitted with the Boltzmann function (see Methods). The half-activation voltage, *V*_half_^Act^, and the slope factor, *s*^Act^, were −44.56 ± 1.82 mV and −2.52 ± 0.16/mV, respectively. In contrast, the voltage dependency of steady-state inactivation (Fig. 1D, upper, see Methods) was also fitted with the Boltzmann function, and *V*_half_^Inact^ and *s*^Inact^ were −102.86 ± 0.69 mV and 3.24 ± 0.26/mV, respectively. The time course of inactivation was characterized by applying long depolarization pulses of 3 s (Fig. 1E, upper). At this time scale, the current traces exhibited slow inactivation after rapid activation. The time constants of activation (τ^Act^, open symbols) and inactivation (τ^Inact^, closed symbols) are shown in Fig. 1E, lower (see Methods): τ^Act^ showed a typical voltage-dependent decrease, whereas τ^Inact^ showed a slightly voltage-dependent increase. These gating features generally reproduce the previously reported KvAP characteristics, featuring voltage-gated channels (14, 27).

### Single-channel current of the KvAP channel in symmetric membrane

**Fig. 2:**
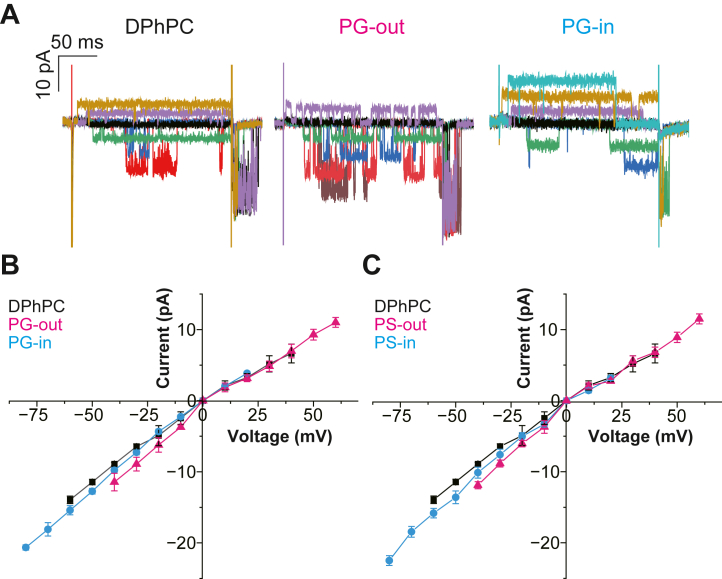
Single-channel current traces and current-voltage curves of the KvAP channel in symmetric and asymmetric membranes. (A) Single-channel currents upon step voltages of −60 to 40 mV and return to −100 mV are shown for symmetric DPhPC (black) (B and C), asymmetric POPG/DPhPC (B), and DPhPC/POPG (C) membranes. For asymmetric membranes, the voltage protocols were shifted by 20 mV hyperpolarization for PS-out and PG-out membranes and 20 mV depolarization for PS-in and PG-in membranes. Single-channel currents were recorded using a Bessel filter with a cutoff frequency of 2 kHz, sampled at 10 kHz.

In parallel with the macroscopic current recordings, single-channel recordings using short depolarization pulses are shown in Figure 2. The channel opened after a delay upon depolarization pulses, and the delay gradually shortened as the membrane potential was depolarized. The tail currents were recorded upon returning to −100 mV, where flicker gating appeared (Fig. 2A left) (40, 41). The single-channel current-voltage curve showed weak inward rectification in the symmetric KCl solution, and the single-channel current conductance was 165.9 ± 33.2 pS at +40 mV (Fig. 2B, black). The single-channel (Fig. 2) behavior of the KvAP channel reproduced previously reported characteristics (24, 42).

In contrast to the symmetric DPhPC bilayer, the KvAP channel was rarely reconstituted into a symmetric POPG and POPS membranes. Ca^2+^ addition does not facilitate channel reconstitution into the CBB (43). As a preliminary step to investigate potential specific interactions and motivate our subsequent asymmetric membrane experiments, we first examined the channel behavior in symmetric membranes with mixed lipid compositions.

### Voltage-dependent gating of the KvAP channel in symmetric mixed membranes

**Fig. 3:**
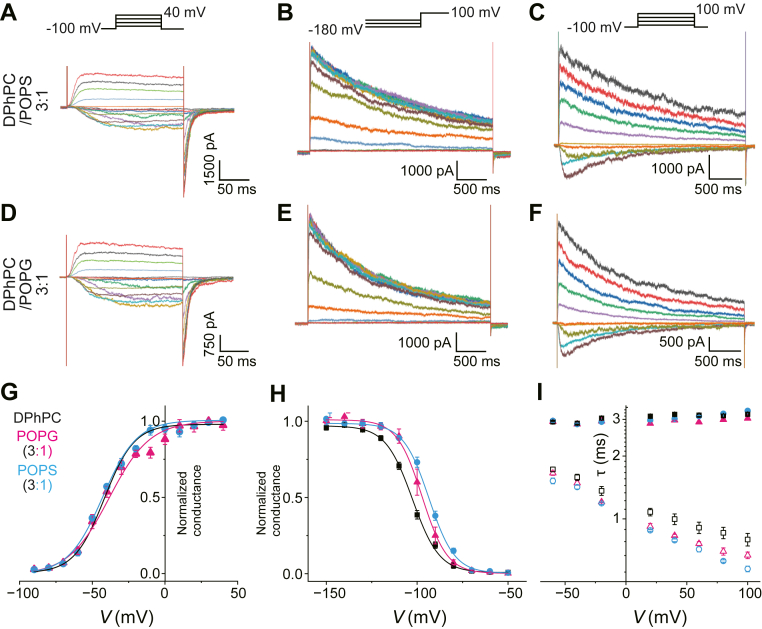
Voltage-gated activities of the KvAP channel in symmetric membranes of mixed lipid composition (DPhPC:POPS or DPhPC:POPG = 3:1). Voltage-dependent activity of the KvAP channel in the mixed symmetric DPhPC:POPS membrane (A-C) and in the mixed symmetric DPhPC:POPG membrane (D-F). A, D: Voltage-dependent activation of the KvAP channel in the symmetric DPhPC:POPS (A) and DPhPC:POPG (D) membranes. The membrane potential was depolarized (−90 to +40 mV) from a holding potential of −100 mV. B, E: Inactivating currents at +100 mV elicited after long hyperpolarized potentials in the mixed symmetric DPhPC:POPG (B) and DPhPC:POPG (E) membranes. C, F: Voltage-dependent slow inactivation of the KvAP channel upon long (3000 ms) depolarizing pulses in the symmetric DPhPC:POPS (C) and DPhPC:POPG (F) membranes. G: *G*-*V* curves for mixed lipid membranes. For the DPhPC:POPS membrane (magenta triangle), *V*_half_^Act^ was −42.45 ± 0.70 mV and *s*^Act^ was −2.23 ± 0.16/mV (N = 5). For the DPhPC:POPG membrane (blue circle), *V*_half_^Act^ was −40.96 ± 1.96 mV and *s*^Act^ was −1.96 ± 0.12/mV (N = 5). The *s*^Act^ value for the DPhPC:POPG membrane was significantly decreased from that of the control membrane. H: Steady-state inactivation curve. For the DPhPC:POPS membrane (magenta), *V*_half_Inact was −93.06 ± 0.99 mV, and *s*^Inact^ was 3.63 ± 0.06/mV (N = 5). For the DPhPC:POPG membrane (blue), the *V*_half_^Act^ was −97.12 ± 2.12 mV, and *s*^Inact^ was 4.31 ± 0.16/mV (N = 5). I: τ–*V* relationship. Voltage-dependent τ^Act^ (open symbols) and τ^Inact^ (closed symbols) are shown for the DPhPC:POPS membrane (magenta), and the DPhPC:POPG membrane (blue).

In the mixed DPhPC:POPS membrane, current traces for the short depolarization pulses, steady-state inactivation, and long depolarization pulses were indistinguishable from those in the DPhPC membrane (Fig. 3A–C). *V*_half_^Act^, *s*^Act^, *V*_half_^Inact^, and *s*^Inact^ were statistically indistinguishable from those of the control membrane (see Fig. 3 legends).

In the DPhPC:POPG membrane, however, the *G*-*V* curve (Fig. 3D, G, blue triangle) showed attenuated voltage-dependence as evidenced by a significantly reduced slope (*s*^Act^ = 1.96 ± 0.12/mV), whereas *V*_half_^Act^ was unaltered (=−40.96 ± 1.96 mV). The other gating properties were negligibly affected (Fig. 3D–F).

To address the sidedness of the POPG effect, experiments were performed on asymmetric membranes.

### KvAP channel activity in asymmetric membranes

In the asymmetric CBB experiments, the left bubble was formed with POPG or POPS, whereas the right bubble was formed with DPhPC (Fig. 1B). Subsequently, a POPG- or POPS-monolayer-lined bubble and a DPhPC-lined bubble were docked to form bilayers. Accordingly, a purely asymmetric CBB membrane of POPG or POPS in the left leaflet and DPhPC in the right leaflet was readily formed (mono-component leaflet membrane; see Methods). The lipid bilayer remained asymmetric as long as the CBB bubbles did not break. All experiments were performed using freshly formed bubbles, and electrophysiological experiments were completed within 30 min, during which the deterioration of asymmetry by lipid flip-flop was not a concern (37).

**Fig. 4:**
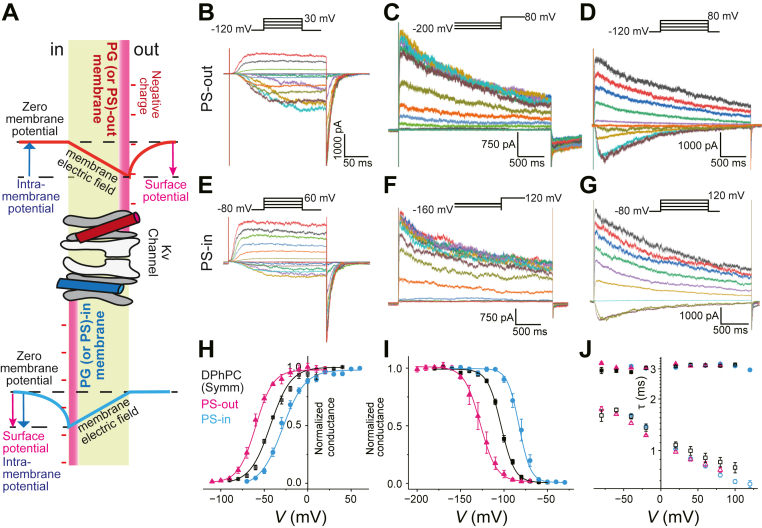
Voltage-gated activities of the KvAP channel in charged asymmetric membranes. A: Relationship between surface potential and intra-membrane potential (*V*_intramemb_). In the presence of a surface potential due to charged lipids, *V*_intramemb_ deviates from *V*_m_ such that *V*_intramemb_ = *V*_m_ - *V*_surf_. Membrane proteins respond to *V*_intramemb_ rather than *V*_m_. The upper and lower halves represent the PS-out and PS-in asymmetric membranes, respectively. The KvAP channel is schematically shown with its PD and VSD domains, where the VSDs are subject to conformational changes under the influence of *V*_intramemb_. B-G: Voltage-gated activities of the KvAP channel in the asymmetric PS-out (B-D) and PS-in (E-G) membranes. B, E: Voltage-dependent activation of the KvAP channel in the asymmetric PS-out (B) and PS-in (E) membranes. The membrane potential was depolarized (−110 to +20 mV for PS-out and −70 to +60 mV for PS-in) from a holding potential (−120 mV for PS-out and −80 for PS-in). C, F. Inactivating currents at +80 mV for PG-out and +120 mV elicited after long hyperpolarized potentials (−200 to −70 mV for PS-out (C) and −160 to −30 mV for PS-in (F)). D, G: Voltage-dependent slow inactivation of the KvAP channel upon long (3000 ms) depolarizing pulses in PS-out (D) and PS-in (G) membranes. H: *G*-*V* curves for the PS-out and PS-in membranes. *V*_half_^Act^ was −61.04 ± 1.70 mV and *s*^Act^ was −2.72 ± 0.23/mV (N = 5) for the PS-out membrane (magenta). *V*_half_^Act^ was −29.93 ± 2.00 mV, and *s*^A^^ct^ was −2.37 ± 0.03/mV (N = 5) for the PS-in membrane (blue). I: Steady-state inactivation curve. *V*_half_^Inact^ was −126.23 ± 4.39 mV and *s*^Inact^ was 2.98 ± 0.14/mV for PS-out (magenta), and *V*_half_^Inact^ was −81.47 ± 2.64 mV and *s*^Inact^ was 3.63 ± 0.23/mV for PS-in (blue) (N = 5). J: τ–*V* relationship. Voltage-dependent τ^Act^ (open symbols) and τ^Inact^ (closed symbols) are shown for the PS-out (magenta), and the PS-in membrane (blue).

In asymmetric membranes, channels are reconstituted with nearly equal probabilities of two opposite orientations. We were able to distinguish these orientations electrophysiologically. Channels that were activated at positive potentials were defined as having their extracellular side facing the charged lipid leaflet (e.g., POPG or POPS) (Fig. 4A). This configuration is termed 'PG-out' or 'PS-out.' Conversely, channels activated at negative potentials were defined as having their intracellular side facing the charged lipid leaflet ('PG-in' or 'PS-in'). For these recordings, the command voltage polarity was reversed.

In charged asymmetric membranes, the surface potential develops on the relevant side of the membrane (Fig. 4A). Consequently, the intra-membrane potential shifts from the transmembrane potential (Fig. 4A) (28). Channel gating responds exclusively to the intramembrane potential rather than the transmembrane potential (44). Consequently, the voltage-dependent gating characteristics (*G*–*V* and steady-state inactivation curves) simply shift in parallel along the voltage axis without changes in the slope when the non-specific surface potential effect predominates. Moreover, this voltage shift should be shared for different voltage-dependent properties, such as activation and inactivation of gating.

### POPS demonstrates a canonical effect consistent with a surface potential

We first examined the gating properties of the KvAP channel in an asymmetric PS-out membrane. In the asymmetric PS-out membrane, the voltage dependency of the channel shifted to more negative potentials, consistent with an electrostatic effect arising from the surface potential of the charged PS leaflet (Fig. 4A). This shift was substantial enough to cause slight channel activation, even at a holding potential of −100 mV. To ensure full channel closure before depolarization, we adjusted the holding potential to −120 mV for these experiments.

Overall, current traces for the short depolarization pulses (Fig. 4B) showed that the inward currents were notably noisier than the outward currents (Fig. 4B) and the channel activated at more negative potentials than the control DPhPC membrane (Fig. 1C), and the mixed DPhPC:POPS membrane (Fig. 3D). The *G*–*V* curve exhibited a substantial negative shift, with *V*_half_^Act^ being −61.04 ± 1.70 mV (Fig. 4G, magenta). Approximately −16 mV of *V*_half_^Act^ was shifted from the control, whereas *s*^Act^ (=−2.72 ± 0.23/mV) was not affected. This gating behavior is characterized by a simple shift in the voltage dependency, indicating that the negative shift in the *G*–*V* curve reflects the magnitude of the potential shift sensed by the channel (Fig. 4A). The steady-state inactivation curve (Fig. 4C) was also shifted negative with *V*_half_^Inact^ of −126.23 ± 4.39 mV and *s*^Inact^ of 2.98 ± 0.14/mV (Fig. 4I), indicating a simple negative shift in the steady-state inactivation. Voltage-dependent activation and inactivation (Fig. 4D) showed similar kinetics (Fig. 4J).

In the asymmetric PS-in membrane, the KvAP channel showed an opposite shift in the voltage dependency of the activation and inactivation gating under the voltage command range shifted in the positive direction by 20 mV (Fig. 4D–F). As the gating parameters shown in Fig. 4H, I, the *G*-*V* curve and the steady-state inactivation curve were shifted in parallel from those in the control membrane, while the slope of the curves was not affected: *V*_half_^Act^ was −29.93 ± 2.00 mV with *s*^Act^ of −2.37 ± 0.03/mV (Fig. 4H) and *V*_half_^Inact^ of −81.47 ± 2.64 mV and *s*^Inact^ of 3.53 ± 0.23/mV (Fig. 4I).

The single-channel current-voltage (*i*–*V*) curves for the PS-out and PS-in membranes are shown in Figure 2C. The single-channel *i*–*V* curve for the PS-out membrane showed a slightly enhanced inward rectification (Fig. 2, magenta), which was expected from the accumulated K^+^ concentration near the highly charged outer membrane. In contrast, the single-channel *i*–*V* curve of the PS-in membrane was indistinguishable from that of the control. This was contrary to the expected increase in the outward current owing to the accumulation of K^+^ near the channel’s intracellular entrance (Fig. 2C, blue). This is likely due to K^+^ permeation through potassium channels, where intracellular K^+^ is mediated by binding to the central cavity before selective permeation through the filter (45, 46).

In summary, POPS, whether in the inner or outer leaflet, induced a simple parallel shift in the voltage-dependence of activation and inactivation gating without altering the slope. In these asymmetric membranes, both the head group and acyl chains of the lipids (POPS vs. DPhPC) differed. Consequently, this behavior is the hallmark of a non-specific electrostatic effect, a purely physical phenomenon consistent with a surface potential. Taken together, these data demonstrate that inner-leaflet POPS exerts a purely physical effect on gating thermodynamics. These results establish a crucial baseline, demonstrating that neither the headgroup nor the acyl chains of POPS engages in specific chemical interactions that alter the channel’s gating thermodynamics.

### POPG in the outer leaflet also induces a shift consistent with a surface potential effect

**Fig. 5:**
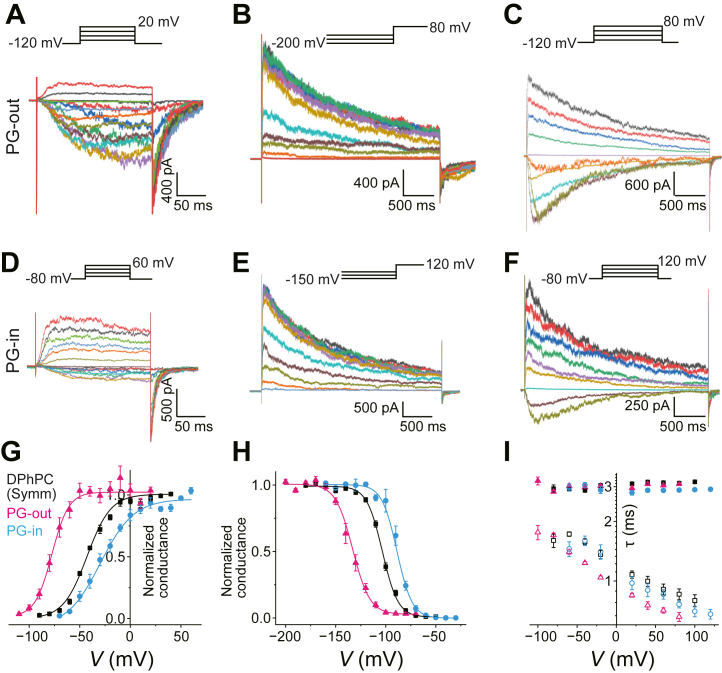
Voltage-gated activities of the KvAP channel in the PG-out and PG-in membranes. A-C: Voltage-gated activities of the KvAP channel in the asymmetric PG-out (A-C) and PG-in (D-F) membranes. A, D: Voltage-dependent activation of the KvAP channel in the asymmetric PG-out (A) and PG-in (D) membranes. The membrane potential was depolarized (−110 to +20 mV for PG-out and −70 to +60 mV for PG-in) from a holding potential (−120 mV for PG-out and −80 for PG-in). B, E: Inactivating currents at +80 mV for PG-out and +120 mV for PG-in elicited after long hyperpolarized potentials (−200 to −70 mV for PG-out (B) and −160 to −30 mV for PS-in (E)). C, F: Voltage-dependent slow inactivation of the KvAP channel upon long (3000 ms) depolarizing pulses in PG-out (C) and PG-in (F) membranes. G. *G*-*V* curves for the PG-out and PG-in membranes. *V*_half_^Act^ was −77.42 ± 1.93 mV and *s*^Act^ of −3.08 ± 0.34/mV (N = 5) for the PG-out membrane (magenta). *V*_half_^Act^ was −27.93 ± 3.13 mV and *s*^Act^ of −1.85 ± 0.13/mV (N = 5) for the PG-in membrane (blue). H: Steady-state inactivation curve. *V*_half_^Inact^ was −132.97 ± 2.19 mV and *s*^Inact^ was 2.88 ± 0.22/mV for PG-out (magenta), and *V*_half_^Inact^ was −87.35 ± 1.52 mV and *s*^Inact^ was 4.59 ± 1.15/mV for PG-in (blue) (N = 5). I: τ–*V* relationship. Voltage-dependent τ^Act^ (open symbols) and τ^Inact^ (closed symbols) are shown for the PG-out (magenta), and the PG-in membrane (blue).

When POPG was confined to the outer leaflet (PG-out), the gating behavior of the channel was qualitatively identical to that observed in PS-out membranes (Fig. 5A–C). It was characterized by a simple parallel shift of the *G*-*V* (*V*_half_^Act^: −77.42 ± 3.08 mV) and steady-state inactivation (*V*_half_^Inact^: −132.97 ± 2.88 mV) curves, with no significant change in their slopes (*s*^Act^: −3.08 ± 0.34/mV and *s*^Inact^: 2.88 ± 0.22/mV) (Fig. 5G, H, Table 1). The only notable difference was the magnitude of the voltage shift, which was more pronounced than that with POPS, suggesting a higher effective charge density for the POPG leaflet (47). These findings confirm that outer leaflet POPG, like POPS, exerts only a canonical surface potential effect (Fig. 5G).

**Table 1:** The gating parameters in the symmetric and asymmetric membranes

Parameters	Symmetric DPhPC	DPhPC:POPS (3:1)	DPhPC:POPG (3:1)	Asymmetric (PS-out)	Asymmetric (PS-in)	Asymmetric (PG-out)	Asymmetric (PG-In)
*V*^Act^	−44.56 ± 1.82	−42.45 ± 0.70	−40.96 ± 1.96	−61.04 ± 1.70	−29.93 ± 2.00	−77.42 ± 1.93	−27.93 ± 3.13
*s*^Act^	−2.52 ± 0.16	−2.23 ± 0.16	−1.96 ± 0.12^a^	−2.72 ± 0.23	−2.37 ± 0.03	−3.08 ± 0.34	−1.85 ± 0.13^a^
*V*^Inact^	−102.86 ± 0.69	−93.06 ± 0.99	−97.12 ± 2.12	−126.23 ± 4.39	−81.47 ± 2.64	−132.97 ± 2.19	−87.35 ± 1.52
*s*^Inact^	3.24 ± 0.26	3.63 ± 0.06	4.31 ± 0.16^a^	2.98 ± 0.14	3.63 ± 0.23	2.88 ± 0.22	4.59 ± 1.15^a^

Data are presented as mean ± SE (N = 5). ANOVA followed by Tukey’s HSD test.

a indicates *P* < 0.01 compared to the symmetric DPhPC control. Statistical significance test was not applied to *V*^Act^ and *V*^Inact^.

### Inner-leaflet POPG induces specific gating modifications beyond the surface potential effect

In the PG-in asymmetric membrane, current traces (Fig. 5D), and the *G–V* curve (Fig. 5G) indicated that the voltage range for the short activation protocol shifted positively, as expected, with *V*_half_ = −27.93 ± 3.13 mV) (Fig. 5G). Surprisingly, *s*^Act^ decreased significantly (−1.85 ± 0.13/mV, *P* < 0.01, Fig. 5G) compared to that in both the PG-out and DPhPC symmetric membranes (Table 1). In contrast, the steady-state inactivation curve shifted to the right (*V*_half_^Inact^ = −87.35 ± 1.52 mV) from those in the PG-out and symmetric membranes, whereas *s*^Inact^ (4.59 ± 1.15/mV) was indistinguishable (Fig. 5H). Moreover, current traces upon long depolarization pulses indicated that the inactivation kinetics were substantially accelerated (Fig. 5F). The τ-*V* curve shows that the time constant for inactivation (τ^*Inact*^) decreased over the positive potentials (Fig. 5I). The τ-*V* curve also shows that the activation kinetics are accelerated. Thus, in contrast to POPS and PG-out membranes, the presence of POPG in the inner leaflet induced multiple profound modifications to the channel gating machinery that could not be explained by surface potential alone: (i) significant attenuation of the *G*-*V* slope, (ii) substantial acceleration of inactivation kinetics, and (iii) acceleration of activation kinetics. These raw observations strongly suggest the presence of specific chemical interactions.

The gating characteristics in all membranes are summarized in Table 1.

### Separating specific chemical interactions from non-specific electrostatic effects

The preceding sections revealed that while most conditions induced simple voltage shifts consistent with an electrostatic effect, the PG-in membrane showed clear signs of specific modifications. To rigorously dissect these specific chemical effects from the non-specific physical effects of charged lipids, we developed a strategy to quantify the latter.

The voltage sensor of the channel does not respond to the externally applied transmembrane potential (*V*_m_) directly, but rather to the intra-membrane potential (*V*_intramemb_) that it experiences within the membrane's electric field (Fig. 4A). In an asymmetric membrane containing charged lipids, *V*_intramemb_ is offset from *V*_m_ by a potential shift, Δ*V*, arising from the surface charge. Consequently, a purely physical, electrostatic effect should manifest as a simple parallel shift of the voltage-dependent gating curves when plotted against the applied *V*_m_, without altering the intrinsic voltage sensitivity (i.e., the slope factor, *s*). This principle leads to two critical predictions for gating parameters governed by thermodynamic equilibria (*G*–*V* and steady-state inactivation curves): (i) the curves should shift in parallel, and (ii) the magnitude of the voltage shift should be equal but opposite in sign for the '-in' versus '-out' configurations (Fig. 4). In this framework, the channel's own voltage-dependent gating serves as the most direct probe of the potential shift, Δ*V*, that it senses.

**Fig. 6:**
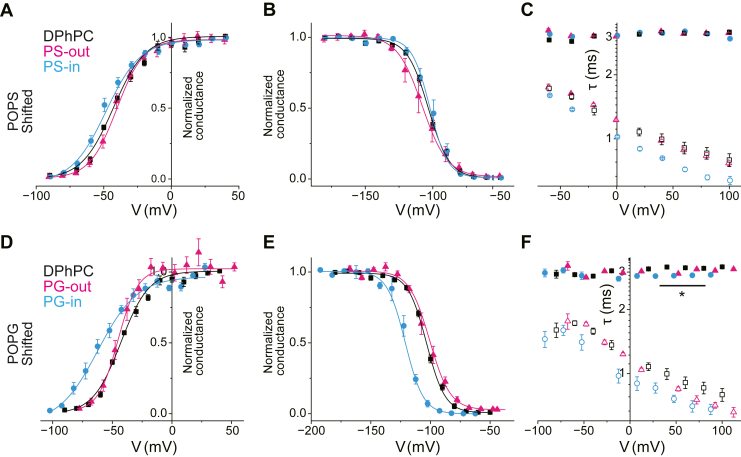
Isolation of specific gating modifications by charged lipids in asymmetric membranes. The voltage-dependent gating curves were drawn by eliminating the voltage shifts of the surface potential. A-C: The gating curves for asymmetric POPS membranes as a function of the *V*_intra-memb_: *G*–*V*_intra-memb_ curves (A), steady-state inactivation curve (B), and τ–*V*_intra-memb_ curve (C). Note the excellent overlap of the curves from PS-out, PS-in, and control conditions, confirming that POPS exerts only a non-specific surface potential effect. Note the excellent overlap of the curves, confirming that POPS exerts only a non-specific surface potential effect on these thermodynamic parameters. D-F: The gating curves for asymmetric POPG membranes as a function of the *V*_intra-memb_: *G*–*V*_intra-memb_ curves (D), steady-state inactivation curve (E), and τ– *V*_intra-memb_ curve (F). In contrast to POPS, the corrected PG-in curve (blue) shows a prominent hyperpolarizing shift and attenuated slope in the *G*-*V* curve (D), and accelerated inactivation kinetics (F), revealing multiple, specific chemical modifications.

Our data from the asymmetric POPS membranes provided perfect experimental validation of this principle. As shown in Table 1, the shifts in *V*_half_ for both the *G*-*V* and steady-state inactivation curves were indeed nearly identical in magnitude but opposite in sign for the PS-in and PS-out conditions, while the slope factors (*s*^Act^ and *s*^Inact^) remained unchanged. This result serves as a robust internal control, confirming that for these thermodynamic parameters, the effect of POPS is exclusively a non-specific, physical one. This validation allowed us to empirically determine the magnitude of the potential shift sensed by the channel due to the POPS leaflet (Δ*V*^PS^) to be 19.43 mV (calculated as the average difference of the *V*_half_^Act^ values for the '-in' and '-out' relative to the control DPhPC configuration) (Fig. 6A–C).

Having quantified Δ*V*^PS^ using the channel itself as a voltmeter, we could then analyze the data as a function of the effective intra-membrane potential (*V*_intramemb_ = *V*_m_ + Δ*V*^PS^). As expected, when replotted against *V*_intramemb_, the *G*–*V* and steady-state inactivation curves from the PS-in, PS-out, and control conditions perfectly overlapped (Fig. 6A, B). This analysis, however, unveiled a subtle but significant specific effect that was previously masked: the activation kinetics (τ^Act^), but not the inactivation kinetics (τ^Inact^), of the PS-in condition were exclusively and significantly accelerated, even after accounting for the electrostatic shift (Fig. 6C). This finding demonstrates that a lipid can exert a purely physical influence on gating thermodynamics while simultaneously inducing a specific chemical effect on gating kinetics.

We then applied the same validated logic to the more complex POPG data set. In contrast to POPS, the raw data for the PG-in condition showed a clear change in the *G*-*V* slope (*s*^Act^), immediately indicating a specific chemical interaction affecting the gating thermodynamics. Therefore, to estimate the physical component of the potential shift for POPG, we relied on the PG-out condition, which, like the POPS conditions, exhibited only a simple parallel shift. This allowed us to determine Δ*V*^PG^ to be 32.36 mV. Re-plotting the PG-in data against its calculated intra-membrane potential (*V*_intramemb_ = *V*_m_ + Δ*V*^PG^) revealed multiple, profound specific chemical modifications (Fig. 6D–F).

In the PG-in membrane, the corrected data indicated that for the activation, (i) the *G-V* curve was significantly shifted negatively, rather than positively, which was uncovered by Δ*V* correction. (ii) The slope of the *G-V* curve was significantly attenuated, and (iii) the activation rates were accelerated. For inactivation gating, (iv) the steady-state inactivation curve was shifted negatively, and (v) the inactivation rate was accelerated. These results demonstrate unequivocally that POPG exerts a complex suite of specific chemical effects on the gating of the KvAP channel, exclusively from the inner leaflet.

## Discussion

Our bottom-up approach using the CBB method with simplified, mono-component asymmetric bilayers has proven to be a powerful strategy for dissecting the complex interplay between membrane lipids and the KvAP channel. We revealed a dramatic leaflet- and headgroup-specificity of charged lipid effects: no specific modifications were observed for lipids in the outer leaflet, whereas inner-leaflet lipids exhibited distinct actions. POPG affected multiple modes of gating, whereas POPS had a much more limited effect, accelerating only the activation kinetics—an effect also shared by POPG. This stark contrast strongly indicates that the specific chemical features of the lipid headgroups dictate their interaction with the inner surface of the channel domains. The central task of this discussion is to integrate these multifaceted findings into a coherent, domain-specific model by contrasting the effects of POPG and POPS.

Here, we integrate the multifaceted actions of inner leaflet lipids to build a domain-specific model of KvAP channel modulation. The key to dissecting the complex effects of POPG lies in contrasting them with the remarkably simple and specific effects of POPS. First, we establish the action of the inner leaflet POPS as a baseline. POPS’s sole specific effect was the acceleration of activation kinetics (τ^Act^) (Fig. 3, Fig. 6I and 6C), without altering any thermodynamic parameters of gating (i.e., *G-V* curve shift or slope) (Fig. 3, Fig. 6G and 6A) or the inactivation kinetics (τ^Inact^) (Fig. 3, Fig. 6H and 6H). This allows us to define the action of the POPS as a purely kinetic modulation of the VSD. This effect is best explained by a mechanism, where POPS interacts with the VSD to lower the energy barrier for its conformational transition, thereby accelerating the rate of charge movement, without changing the relative stability of the down and up states of the charge movements. Acceleration of activation kinetics was also observed with inner leaflet POPG, suggesting a shared mechanism of VSD modulation. This common effect, observed with two different anionic lipids, suggests that the interaction is likely mediated by a general electrostatic interaction between the negatively charged headgroups and the VSD, which facilitates its outward movement. The fact that POPS exerts only this kinetic effect, in contrast to the intimate chemical interactions of POPG with other domains (discussed below), further supports the notion that this VSD-kinetic modulation is a more general phenomenon.

Beyond this shared kinetic effect, POPG exerted three additional, profound modifications, all of which can be attributed to its specific interactions with the PD and its linker with the VSD. The most conclusive evidence for a POPG-PD interaction is the acceleration of the inactivation kinetics (τ^Inact^) (Fig. 6F). Inactivation gating involves conformational changes in the selectivity filter located at the outer part of the PD (48). For an inner leaflet lipid to influence this process, its binding to the inner half of the PD must induce an allosteric change that is transmitted across the PD structure, ultimately lowering the energy barrier for the open-to-inactivated state transition. The absence of this effect with POPS unequivocally demonstrates that this is a chemically specific interaction that is unique to POPG.

This intimate POPG-PD interaction also provides the most rational explanation for the negative shift in the *G*-*V* curve after correcting for electrostatic effects (Fig. 6D), indicating that the open state was thermodynamically stabilized relative to the closed state. Because the final open-closed equilibrium is determined by the PD's activation gate, this stabilization is a direct consequence of POPG's interaction with the inner half of the PD, which undergoes substantial conformational changes upon gating (49). The concurrent negative shift of the steady-state inactivation curve (Fig. 6E) can be largely understood as a thermodynamic consequence of this open-state stabilization.

Finally, the most prominent finding of this study was the POPG-induced decrease in the *G*-*V* slope (*s*^Act^) (Fig. 6D), a hallmark of impaired electromechanical coupling (50, 51). This indicates that the conformational change in the activated VSD is less efficiently translated into the opening of the PD's activation gate. Given that the S4-S5 linker, the physical conduit for this coupling, is located at the inner membrane surface, it is highly plausible that POPG specifically interacts with this linker or the VSD-PD interface, thereby disrupting its function.

In summary, our data support a multi-site interaction model where inner leaflet POPG exerts its effects through at least two distinct mechanisms: (i) a general kinetic modulation of the VSD, shared with POPS, that accelerates activation, and (ii) a suite of specific chemical interactions with the PD and the VSD-PD linker that stabilize the open state, accelerate inactivation, and impair electromechanical coupling. The molecular basis of these specific interactions warrants further functional and structural studies (52, 53, 54).

Our findings gain further significance when contrasted with those of other well-studied lipids. Notably, phosphatidic acid (PA) from the inner leaflet induces a large positive shift in the *G*-*V* curve (28). This effect, opposite to that of POPG, powerfully underscores that the fine chemical structure of the headgroup, not merely its negative charge, dictates the functional outcome of lipid modulation. Even more striking is the comparison with the signaling lipid PIP_2_, which is known to be an essential cofactor for facilitating VSD-PD coupling in channels such as KCNQ1 (18, 55, 56). Our discovery that POPG, a common structural lipid, acts as an inhibitor of this same process reveals a new dimension of lipid regulation. It suggests that the electromechanical coupling efficiency is not static but can be dynamically tuned across a spectrum—from facilitation to attenuation—by the local concentration of different annular lipids.

In conclusion, this study demonstrates that a common structural lipid, POPG, acts as a highly specific, multi-target modulator of Kv channels, but only from the inner membrane leaflet. The discovery that a bulk lipid can function as a negative regulator of VSD-PD coupling provides a crucial conceptual advance, suggesting that the fidelity of voltage sensing is under constant regulation by the surrounding lipidome. While we have used KvAP as a robust model system, its non-domain-swapped architecture (57, 58, 59, 60, 61, 62), which creates a unique intra-subunit VSD-PD interface, likely provides the structural scaffold for these specific interactions and serves as an important point of comparison for future studies on domain-swapped channels (63). Future studies combining mutagenesis of the proposed interaction sites with structural approaches will be essential to validate the proposed multi-site interaction model.

## Data availability

The authors declare that all supporting data and methods are available within the article. The data that support the findings of this study are available from the corresponding author upon reasonable request.

## Conflict of Interest

The authors declare that they do not have any conflicts of interest with the content of this article.
